# Early Flare-Ups of Myasthenia Gravis After Thoracoscopic Thymectomy in a Patient Recently Receiving BNT162b2 mRNA COVID-19 Vaccination

**DOI:** 10.7759/cureus.21571

**Published:** 2022-01-24

**Authors:** Permphan Dharmasaroja

**Affiliations:** 1 Neurology, Chakri Naruebodindra Medical Institute, Faculty of Medicine Ramathibodi Hospital, Mahidol University, Samut Prakan, THA

**Keywords:** mrna vaccine, vaccination, myasthenia gravis exacerbation, thymectomy, myasthenia gravis, covid-19

## Abstract

Myasthenia gravis (MG) is an autoimmune disorder characterized by abnormal neuromuscular transmission. The thymus is believed to play a key role in the pathogenesis of MG, and thymectomy has been an optional treatment for the disease. Relapse of MG after thymectomy has been reported. Exacerbations and new onset of MG following COVID-19 vaccination have also been documented. This report presents a case of a stable MG patient with recent COVID-19 vaccination experiencing flare-ups of symptoms shortly after video-assisted thoracoscopic (VATS) thymectomy. A 31-year-old female received the second dose of the BNT162b2 mRNA COVID-19 vaccine eight days before thymectomy and developed flare-ups of symptoms four days after the surgery. Although the substantial link between MG exacerbations post-thymectomy and pre-thymectomy COVID-19 vaccination cannot be concluded, this observation warrants further research.

## Introduction

Myasthenia gravis (MG) is an autoimmune neurologic disease characterized by abnormal neuromuscular transmission. Antibodies against acetylcholine receptors (AChRs), muscle-specific kinase (MuSK), and low-density lipoprotein receptor-related protein 4 (LRP4) are the predominant autoantibodies in MG [1]. The disease is found more frequently in women at younger ages between 20 and 30 years, and it affects both sexes equally in advanced ages [2]. The thymus is believed to play an important role in the pathogenesis of MG, and it exhibits morphological changes as lymphofollicular hyperplasia, thymoma, or thymic atrophy [3].

Surgical removal of the thymus has been an alternative treatment for MG. A prospective, randomized, controlled clinical trial has shown conclusive evidence that thymectomy benefits patients with non-thymomatous MG and reduces the requirement for immunosuppressive therapy [4]. Relapse of MG after thymectomy has been reported. However, the study on the prevalence and the factors associated with recurrence following thymectomy remains scant. A systematic review of observational studies demonstrated that 18% of MG patients who had thymectomy experienced recurrence during follow-up [5]. Almost a fifth of the patients developed MG recurrence after an average duration of about 4.7 years post-thymectomy. Risk factors for recurrence include older age (>35-60 years old), male sex, disease severity (>class II), thymoma, longer duration of MG before surgery, and an ectopic thymic tissue [5].

New onset of MG has been reported in two individuals after receiving the second dose of the BNT162b2 mRNA COVID-19 vaccine [6]. Exacerbations of the disease in a patient with stable MG following the second dose of the mRNA-1273 COVID-19 vaccine have also been reported [7]. It is noteworthy that all patients were males, older than 70 years, and developed clinical symptoms of MG within seven days after COVID-19 vaccination, with two-thirds of them being severe. There is also a report on 21 MG patients receiving inactivated vaccines and one MG patient receiving a recombinant subunit vaccine [8]. Of the 21 MG patients receiving inactivated vaccines, 20 patients did not present MG symptoms worsening within four weeks of COVID-19 vaccination, and six of them already had undergone thymectomy. For the two patients, one 51-year-old male with an inactivated vaccine and one 52-year-old female with a recombinant subunit vaccine, who reported slight symptom-worsening at 6-10 days after vaccination, both patients had thymoma and underwent thymectomy and were in the stable stage of the disease prior to COVID-19 vaccination.

The rapid advancement of COVID-19 vaccines has prompted concerns regarding vaccine safety and efficacy in immunocompromised individuals, such as those with MG. Whether vaccination increases the risk of MG symptoms worsening remains unknown. It's challenging to estimate the safety of mRNA, DNA, and viral vector COVID-19 vaccines in MG because of their novelty. This paper reports a temporal relationship between the BNT162b2 COVID-19 vaccination and symptom exacerbations shortly after thymectomy in a young female MG patient.

## Case presentation

**Figure 1:**
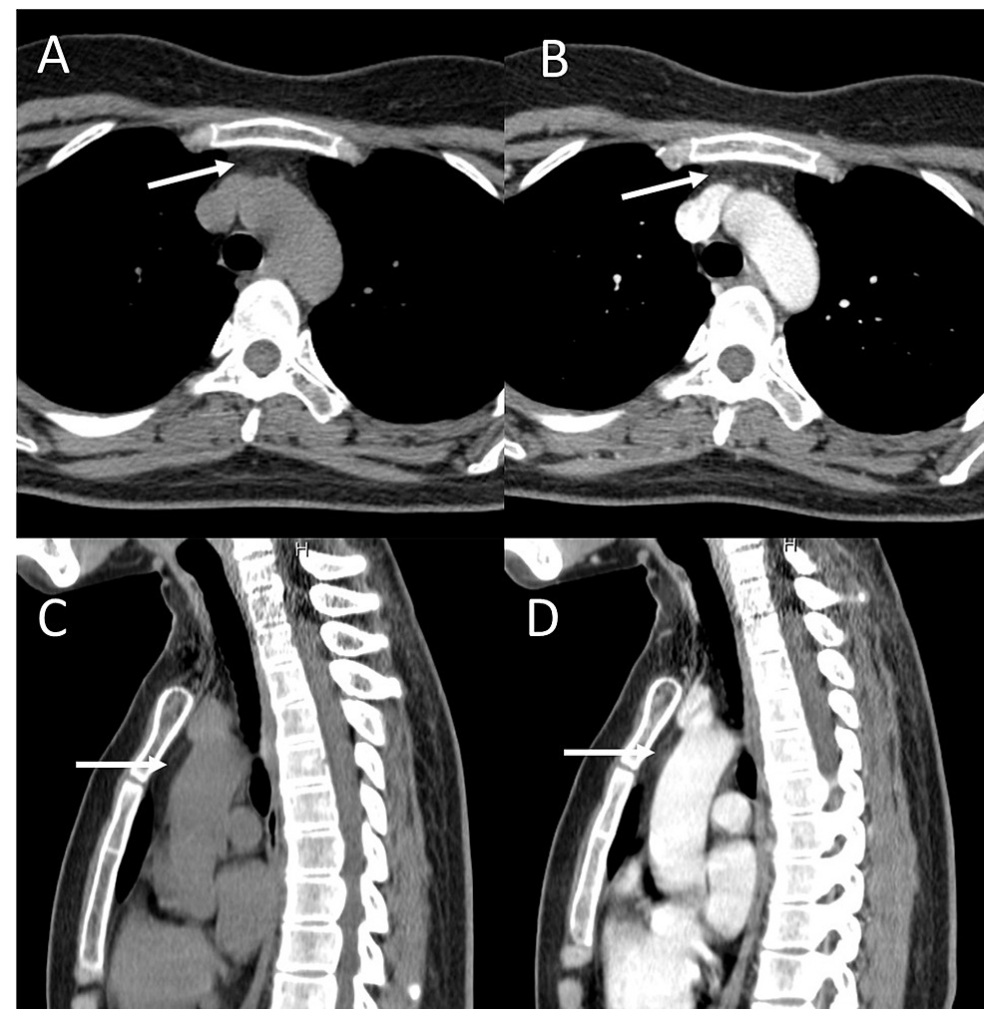
MDCT of the chest (A) Axial non-contrast, (B) axial with contrast, (C) sagittal non-contrast, and (D) sagittal with contrast views show prominent soft tissue without abnormal enhancement at mid-superior mediastinum in the prevascular space anterior to the great vessels (arrow). MDCT, Multidetector computed tomography.

A 31-year-old Thai female first presented with fatigable bilateral ptosis and proximal muscle weakness 10 months ago and was diagnosed with MG. The symptoms were partially improved with rest. Her blood test was negative for anti-AChR antibodies. Thymic mass screening with chest multidetector computed tomography (MDCT) disclosed prominent soft tissue at mid-superior mediastinum without abnormal enhancement, measured about 0.98 cm x 2.29 cm in its greatest transverse dimension (Figure 1).

**Figure 2:**
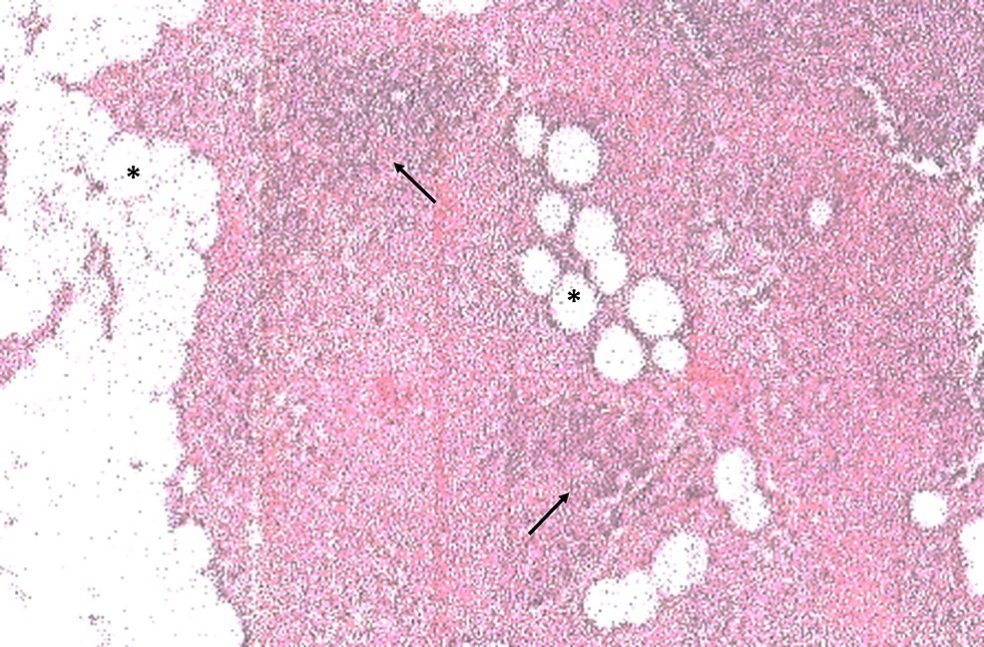
Histological examination shows involuting thymic tissue The section shows indistinct corticomedullary junction, decreased cortical lymphocytes (arrows), and adipocyte infiltration (asterisks).

She has been maintained on 5-mg prednisolone three tablets every other day and 60-mg pyridostigmine two tablets thrice a day, with stable symptoms at the Myasthenia Gravis Foundation of America (MGFA) class IIa. She received the first and second doses of the BNT162b2 COVID-19 vaccine approximately four weeks and eight days prior to the scheduled thymectomy. The preoperative nasopharyngeal and throat swab RT-PCR test for SARS-CoV-2 RNA was negative. Thymectomy was performed via the lateral approach using right-sided video-assisted thoracoscopic surgery (VATS) under general anesthesia with endotracheal intubation. The pathological result revealed lobulated yellow tissue containing islands of involuting thymic tissue and foci of hemorrhage (Figure 2).

Perioperative hydrocortisone was intravenously administered and tapered off within 24 hours. The usual dosages of prednisolone and pyridostigmine were then resumed. The patient was discharged three days after surgery, with her MG symptoms being stable as previously. However, on the next day (12 days after the second dose of the COVID-19 vaccine), the patient reported more weakness on her hips, thighs, and shoulders. Fatigable ptosis was more frequent with occasional diplopia. Her voice was faded away when talking longer, and she felt tired more easily. Laboratory investigation ruled out possible infections, and serum potassium was normal. Prednisolone was gradually increased to 20 mg daily and pyridostigmine was increased to two tablets four times a day. At a two-week follow-up, the patient reported improvement of muscle strength, the disappearance of diplopia, a normal voice, and having regular daily activities.

## Discussion

The flare-ups of MG symptoms after thymectomy in this patient suggested the temporal relationship with the second dose of the mRNA-1273 COVID-19 vaccine. It is well known that infections are closely associated with the onset of MG and are the most common contributor to MG progression [9]. Numerous studies have observed the relationship between active COVID-19 infections and MG exacerbations. MG patients with COVID-19 have a higher risk of severe pneumonia and myasthenia crises as well as a higher mortality rate [10]. In order to protect MG patients against COVID-19, it is very crucial to vaccine them. In this patient, she received the COVID-19 vaccine within two weeks before MG exacerbation, and the septic workup was negative. Therefore, it is likely that the vaccination was most likely to be responsible for the patient's exacerbation. Worsening of MG after thymectomy has been reported. A meta-analysis showed that history of post-surgery myasthenia crisis, thymoma, generalized MG, bulbar symptom, and concomitant complication is the risk factor of post-surgery myasthenia crisis [11]. Although post-surgery myasthenia crisis has been reported, this condition is usually related to respiratory muscle paralysis due to prolonged or repeated mechanical ventilation. However, none of these factors, except generalized MG, was found in the patient in this report.

It is uncertain whether thymectomy performed soon after COVID-19 vaccination contributed to MG aggravation. Vaccines or adjuvants could theoretically cause or aggravate autoimmune illnesses by triggering a generalized autoimmune response, resulting in multiple autoantibodies, and reproducing human autoimmune diseases [12]. Thymic injury during thymectomy may induce an inflammatory state. Soluble factors such as interleukin (IL)-6 released from the injured thymus may stimulate acute phase responses and immune reactions [13]. It is plausible that COVID-19 vaccine-related cytokine storm could also constitute a common denominator for MG exacerbation in the post-thymectomy patient in this report, who is young, female, and non-thymomatous. Although the strong association of MG exacerbations post-thymectomy with pre-thymectomy COVID-19 vaccination in this case report cannot be concluded, this observation warrants further systematic studies.

Due to mild flares, doses of prednisolone and pyridostigmine were increased to control the patient’s symptoms, with considerable improvement. A previous study showed that prednisolone dose-dependently inhibited the release of pro-inflammatory mediators such as tumor necrosis factor α (TNF-α), IL-6, and IL-8 while enhancing the release of the anti-inflammatory cytokine IL-10 [14]. In critically ill COVID-19 patients, seven of 10 patients had stable or decreased plasma IL-6 levels after steroid administration [15]. These data suggest the beneficial roles of corticosteroid therapy in cytokine storm-related illness.

## Conclusions

This case report provides clinical observation on a temporal relationship between the BNT162b2 COVID-19 vaccination and MG flare-ups shortly after thymectomy in a young female patient. Guidance on the care of patients with MG during the COVID-19 pandemic has been issued by a variety of professional groups. Unless there is a specific contraindication, COVID-19 vaccination is suggested for patients with MG. Although there is no strong evidence that COVID-19 vaccines induce cytokine storm, decisions on timing for thymectomy or other major scheduled surgery should be made on a case-by-case basis as inflammatory cytokines released during thymic injury and possibly as a result of COVID-19 vaccines or adjuvants may aggravate the symptoms of MG. Several types of COVID-19 vaccines, including mRNA, DNA, viral vector, protein-based, and inactivated vaccines, have been developed in the SARS-CoV-2 virus pandemic situation. The safety profile and efficacy of these COVID-19 vaccines in MG patients should be further investigated.
